# Supplementary material for: Nutrition, Flavor, and Microbial Communities of Two Traditional Bacterial Douchi from Gansu, China
Source: Foods. 2024 Nov 4;13(21):3519. doi: 10.3390/foods13213519 (PMC11545533; doi:10.3390/foods13213519)
Supplement: Supplementary file 1 [file foods-13-03519-s001.zip › foods-3265416-supplementary.pdf]

Table S1. Comparison of the essential amino acid composition in Longnan and Qingyangdouchi with WHO/FAO recommended pattern （g /100g）.

Types	Threonine	Lysine	Isoleucine	Leucine	Phenylalanine	Valine
Recommended standard pattern	4.00	5.50	4.00	7.00	6.00	5.00
Longnandouchi average value	0.58	0.82	0.80	1.72	1.19	0.74
Qingyangdouchi average value	0.24	0.60	0.43	0.91	0.66	0.45

Table S2. Analysis of RAA, RC and SRC in Longnan and Qingyangdouchi.

Essential amino acid	Ratio of amino acid （RAA）		Ratio coefficient of amino acid（RC）		Score of ratio coefficient of amino acid（SRC）	
	LN douchi	QY douchi	LN douchi	QY douchi	LN douchi	QY douchi
Threonine	0.15	0.06	0.80	0.59	79.51	78.51
Lysine	0.15	0.11	0.88	1.08		
Isoleucine	0.20	0.11	1.18	1.06		
Leucine	0.25	0.13	1.45	1.29		
Phenylalanine	0.20	0.11	1.17	1.09		
Valine	0.15	0.09	0.82	0.89		
